# Multi-Scale Statistical Correlation between Morphological Anisotropy and Optical Response in Chemically Deposited AZO Nanorods

**DOI:** 10.1021/acsomega.6c01472

**Published:** 2026-05-21

**Authors:** Fatma Sarf, Mehmet Bayırlı

**Affiliations:** † Çan Vocational School, Çanakkale Onsekiz Mart University, Çanakkale 17100, Turkey; ‡ Physics Department, Balıkesir University, Balıkesir 10145, Turkey

## Abstract

This work presents a multiscale statistical framework that quantitatively correlates the anisotropic morphology of solution-grown aluminum-doped zinc oxide (AZO) nanorods with their optical response. AZO nanorods were synthesized via chemical bath deposition on ZnO nanoflower seed layers and systematically characterized using field-emission scanning electron microscopy (FE-SEM). Advanced image processing combined with circular statistical analysis was employed to extract three complementary morphological descriptors: (i) the log-normal distribution parameters of the nanorod aspect ratio (AR), (ii) a scale-dependent critical exponent γ derived from power-law scaling, and (iii) the orientation concentration parameter κ obtained from the von Mises distribution. The results reveal a pronounced scale-dependent growth regime, with γ increasing from 1.307 at 0.1 μm to 3.881 at 30 μm, indicating a transition from diffusion-limited atomic-scale growth to fractal-like aggregation at larger length scales. The nanorods exhibit strong vertical alignment (mean tilt angle θ ≈ 89.0° ± 3.7°), while significant in-plane orientational disorder is observed, as reflected by a reduction of κ from 1.721 to 1.229 at finer spatial resolutions. Optical characterization shows a blue-shifted absorption edge (∼370 nm) and defect-related photoluminescence, both of which are shown to correlate directly with the quantified morphological anisotropy. By linking multiscale structural statistics (γ, κ, and log-normal AR parameters) to optical functionality, this framework provides physically grounded design guidelines for engineering the optoelectronic performance of AZO nanostructures through controlled morphological tuning.

## Graphical abstract

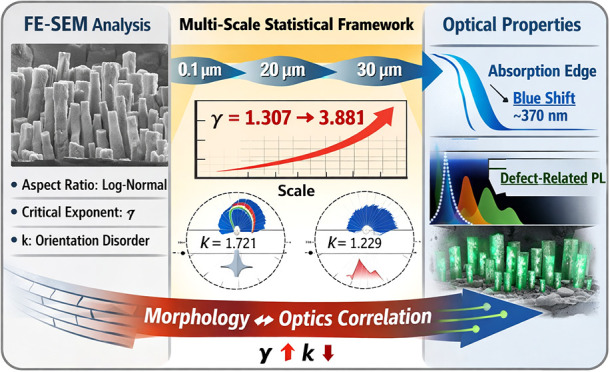

## 1 Introduction

II–VI group semiconductor materials play a crucial role in a wide range of photonic and optoelectronic device applications, including optical waveguides, photodetectors, sensors, light-emitting diodes, and lasers, owing to their wide band gap tunability (from 0 to ∼4 eV), capability to form binary, ternary, and quaternary compounds, and their ability to facilitate efficient charge separation.^1,2^ Following the successful demonstration of blue–green laser emission from ZnS-based structures and the commercial production of CdTe-based solar cells, these materials have attracted significant scientific and technological interest.^3^ Among them, ZnO has emerged as a particularly promising semiconductor due to its wide direct band gap (∼3.37 eV), high refractive index, rich defect chemistry (especially zinc interstitials, Zni, and oxygen vacancies, Ov), and natural abundance.^4^ Recent advances in ZnO-based photonic and optoelectronic devices have been enabled through controlled doping, dimensional engineering (from 0D to 3D nanostructures), and optimization of metal–semiconductor contacts.^5^

One-dimensional (1D) nanorod structures, in which elongated nanostructures are aligned either vertically or horizontally with respect to the substrate, have attracted considerable attention due to their anisotropic physical properties.^6^ In particular, nanorods exhibit unique plasmonic and optical performance that are highly relevant for sensing and imaging applications.^7^ Their high surface-to-volume ratio further makes them attractive for catalytic processes and drug delivery systems by enhancing chemical reactivity.^8^ The anisotropic nature of nanorods gives rise to orientation-dependent optical absorption, photoluminescence, and directional charge transport characteristics. Consequently, tailoring the morphology and orientation of ZnO nanorods through controlled synthesis routes has become a key strategy for developing functional photonic devices, as the optical properties of dielectric nanorods are strongly governed by their structural and compositional features.^9^ Moreover, aluminum (Al^3+^) doping represents an effective strategy for tailoring the structural and optical properties of ZnO due to the close match between the ionic radius of Al^3+^ (0.57 Å) and that of Zn^2+^ (0.74 Å), as well as their similar electronegativity values on the Pauling scale (1.61 for Al and 1.65 for Zn). This compatibility facilitates substitutional incorporation of Al into the ZnO lattice, leading to the formation of aluminum-doped zinc oxide (AZO). Previous studies have shown that moderate Al doping can significantly influence the microstructural and optical characteristics of ZnO films. For instance, Chowdhury et al. reported that AZO films with 4% Al content exhibited reduced particle size and suppressed aggregation, whereas higher doping levels (6% and 8%) resulted in increased surface roughness and reduced crystallinity.^10^ Similarly, an increase in optical band gap energy from 3.16 to 3.25 eV upon Al incorporation has been observed, attributed to carrier concentration effects.^11^ The influence of Al doping and processing conditions on the optoelectronic performance of AZO has also been widely reported. Arzaee et al. demonstrated that three AZO layers with an individual thickness of 80 nm yielded an optimal figure of merit (FOM) of 1.42 × 10^–9^ Ω^–1^.^12^ Khan et al. showed that sol–gel spin-coated AZO films with 2% Al content, annealed at 500 °C, exhibited a high optical band gap of 3.67 eV, a transmittance of 84.19%, and a low volume resistivity of 2.05 Ω·cm.^13^ Furthermore, controlled hydrothermal growth of nanorods enabled simultaneous achievement of low sheet resistance on the order of 10^1^ Ω/sq and high average optical transmittance exceeding 80%.^14^ Additional postdeposition treatments, such as γ (γ) irradiation (1–4 kGy) during RF magnetron sputtering, have been shown to reduce the refractive index, increase defect density, and induce a red shift in the optical band gap from 3.43 to 3.25 eV.^15^

Among the various synthesis techniques, chemical bath deposition (CBD) is widely regarded as a simple, low-cost, and environmentally friendly method that does not require sophisticated vacuum systems or complex instrumentation. Owing to these advantages, CBD has been extensively employed for the fabrication of AZO thin films with tunable structural, optical, and electrical properties. For example, Kumar et al. reported that AZO films deposited via CBD exhibited a maximum optical transmittance of 87% and a minimum electrical resistivity of 8.53 × 10^–3^ Ω·cm.^16^ In our previous work, AZO films grown by CBD using a powder Al source demonstrated the highest ammonia (NH_3_) gas sensing response, indicating that the Al dopant source plays a critical role in controlling the surface-to-volume ratio and defect-related surface chemistry.^17^ Taken together, these studies highlight that both the Al dopant characteristics and the CBD process parameters have a pronounced influence on surface morphology, defect speciation, and, consequently, the functional properties of films.

AZO films were deposited by sol–gel dip-coating process and the average crystallite sizes decreased from 28 to 25 nm and optical band gap increased from 3.09 to 3.26 eV when Al doping increases.^18^ In another study, (Al, In) doped ZnO thin films have been grown on glass substrate at 400 °C by the USP technique and AZO average grain size was found of 12 nm while the optical band gap has not changed with Al-doping.^19^ Sol–gel dip coating applied ZnO:Al/p-Si diode properties were investigated and calculated according to the thermionic and Chueng models.^20^

However, studies addressing the statistical influence of surface nanorod distributions on nucleation density and growth kinetics of ZnO-based structures remain limited, particularly under conditions of heterogeneous nanorod arrangements. A comprehensive description of the anisotropic properties of Al-doped ZnO surfaces requires the simultaneous evaluation of both the polar (orientation) angle (θ) and the azimuthal angle (φ). It is well established that controlled clustering in metal nanorod arrays can lead to strongly enhanced optical and field-related phenomena, including pronounced field emission characteristics.^21^ Despite numerous qualitative investigations of AZO nanorod morphology, a quantitative multiscale statistical framework that directly correlates anisotropic growth dynamics with optical response is still lacking, especially for chemically deposited systems.^22^ In this study, we address this gap by performing a comprehensive statistical analysis of chemically deposited AZO nanorod films aimed at elucidating their optical behavior. Scaling theory and photometric analysis are employed to characterize nanorod morphology across macro-, micro-, and nanoscopic length scales using field-emission scanning electron microscopy (FE-SEM) images. This multiscale approach enables direct correlation between surface anisotropy, growth statistics, and optical response, providing new insight into structure–property relationships in AZO nanorod systems.

## 2 Materials and Methods

Details of the experimental procedure for AZO film deposition on nanoflower-like ZnO seed layers, as well as the structural and NH_3_ gas sensing properties of the samples, have been reported in our previous studies.^17,23^ A schematic diagram was exhibited in Figure 1. Surface morphology was examined using field-emission scanning electron microscopy (FE-SEM, JEOL JSM-7100F). Optical absorption measurements were performed using a UV–visible spectrophotometer (PerkinElmer Lambda 2S) over the wavelength range of 300–900 nm. Photoluminescence (PL) spectra were acquired using a PerkinElmer LS 55 molecular fluorometer coupled with an Andor Solis SR 500i-BL spectrometer, with a Nd:YLF laser (λ = 349 nm) employed as the excitation source. All characterization measurements were carried out at room temperature.

**Figure 1:**
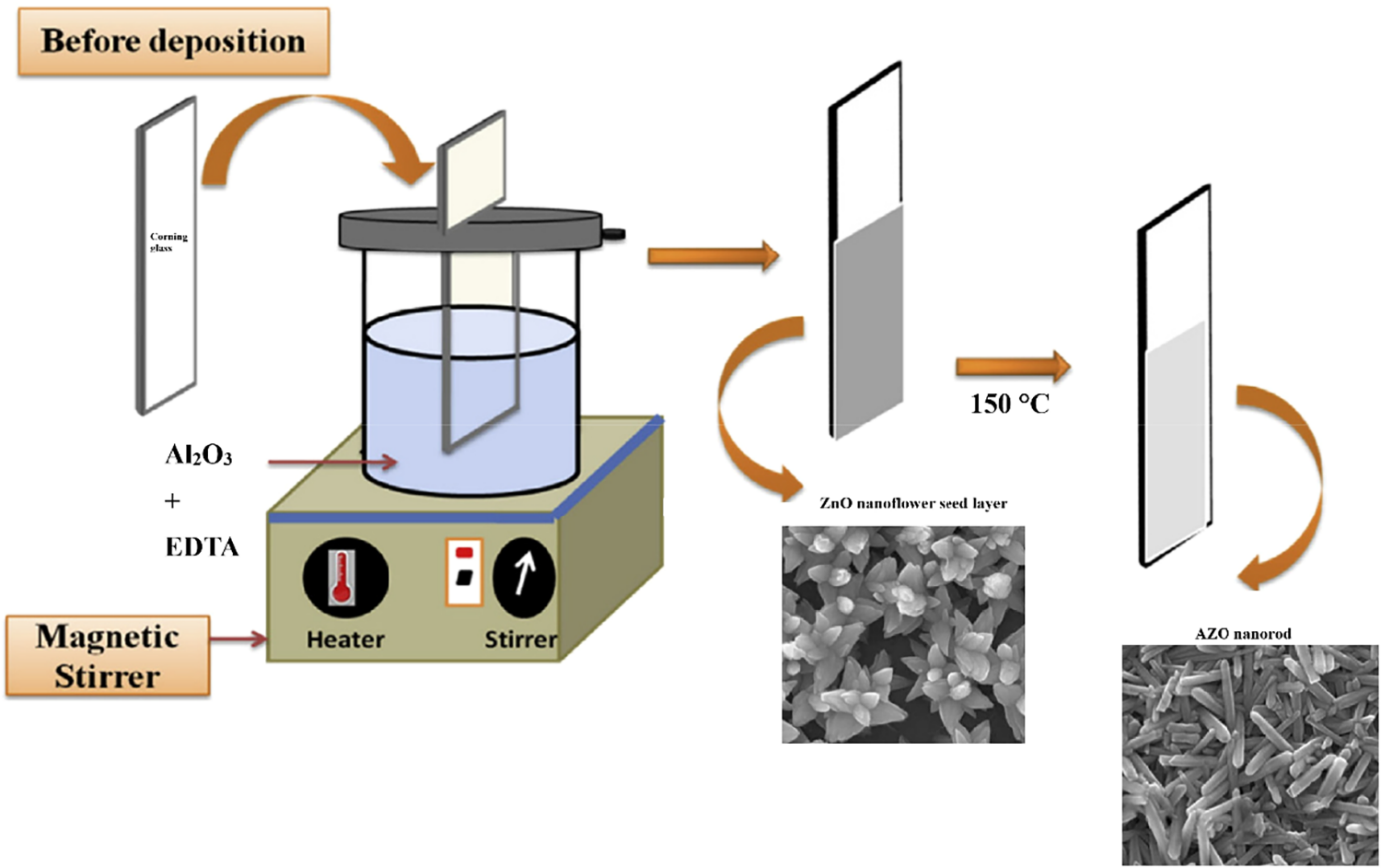
Schematic diagram of AZO nanorod synthesis process by chemical bath deposition.

Surface morphology was studied using FE-SEM images acquired at multiple length scales of 0.1 μm, 10 μm, 20 μm, and 30 μm. Image calibration was performed based on the scale bar, yielding a spatial resolution of 30 μm/3000 pixels, corresponding to 0.01 μm per pixel. Following image acquisition, the effective scaling factor was determined to be 1160.31 pixels per μm. Image processing and statistical analysis were carried out using open-source Python-based software. Initially, grayscale images were normalized and subjected to contrast enhancement. Statistical filtering was subsequently applied to suppress background noise and reduce contrast fluctuations, followed by Otsu’s adaptive thresholding method^24^ to convert the images into binary form. Morphological operations, including the removal of small isolated objects and hole filling within individual nanorods, were performed based on the observed image features. To separate merged or overlapping nanorods, a watershed segmentation algorithm was applied. Finally, numerical postprocessing and data corrections were conducted to enable reliable statistical evaluation of the nanorod morphology.

The FE-SEM images were processed using a custom Python script (Python 3.9) incorporating OpenCV (cv2, v4.8) for image thresholding and morphological operations, scikit-image (v0.21) for median filtering and watershed segmentation, NumPy and SciPy for numerical computations and statistical fitting, and Matplotlib for visualization. Grayscale images were initially normalized to enhance contrast and subjected to median filtering with a 3 × 3 pixel kernel to suppress noise. Automated binarization was performed using Otsu’s thresholding method, with threshold values determined independently for each image scale to account for intensity variations. Morphological operations, including erosion and dilation with a 3 × 3 circular structuring element, were applied to remove small artifacts and fill holes within individual nanorods. To separate overlapping or agglomerated nanorods, a watershed segmentation algorithm based on distance transformation was employed. Objects with an area smaller than 50 pixels were excluded to minimize noise contributions. Each segmented nanorod was subsequently labeled, and its geometric parameters—including length, width, and orientation—were extracted. The aspect ratio (AR) was defined as the ratio of the major axis length to the minor axis width. Nanorod orientation vectors were determined using principal component analysis (PCA) applied to each segmented region, enabling reliable extraction of both tilt and in-plane orientation angles for subsequent statistical evaluation.

## 3 Results and Discussion

### 3.1 Aspect Ratio of AZO Nanorods

Al-doped ZnO (AZO) nanorods were synthesized using the chemical bath deposition method. To capture the multiscale morphological architectures from micro- to macroscopic length scales, FE-SEM images were acquired from different regions of the sample at four characteristic magnifications: 0.1 μm, 10 μm, 20 μm, and 30 μm, hereafter referred to as Sample 1, Sample 2, Sample 3, and Sample 4, respectively. A representative high-magnification image at the 0.1 μm scale is presented in Figure 2(a). Figure 2(b) shows the same image in two complementary representations, while Figure 2(c) illustrates the identification of distinct elongated features, highlighted in different colors, yielding a total of 527 statistically resolved clusters. Figure 2(d) presents the corresponding object detection results, in which 30 individual anisotropic units were segmented and examined. A schematic depiction of the feature length and width used for aspect ratio determination is also included in Figure 2(d). All relevant morphological characteristics are indicated by orange arrows for clarity.

**Figure 2:**
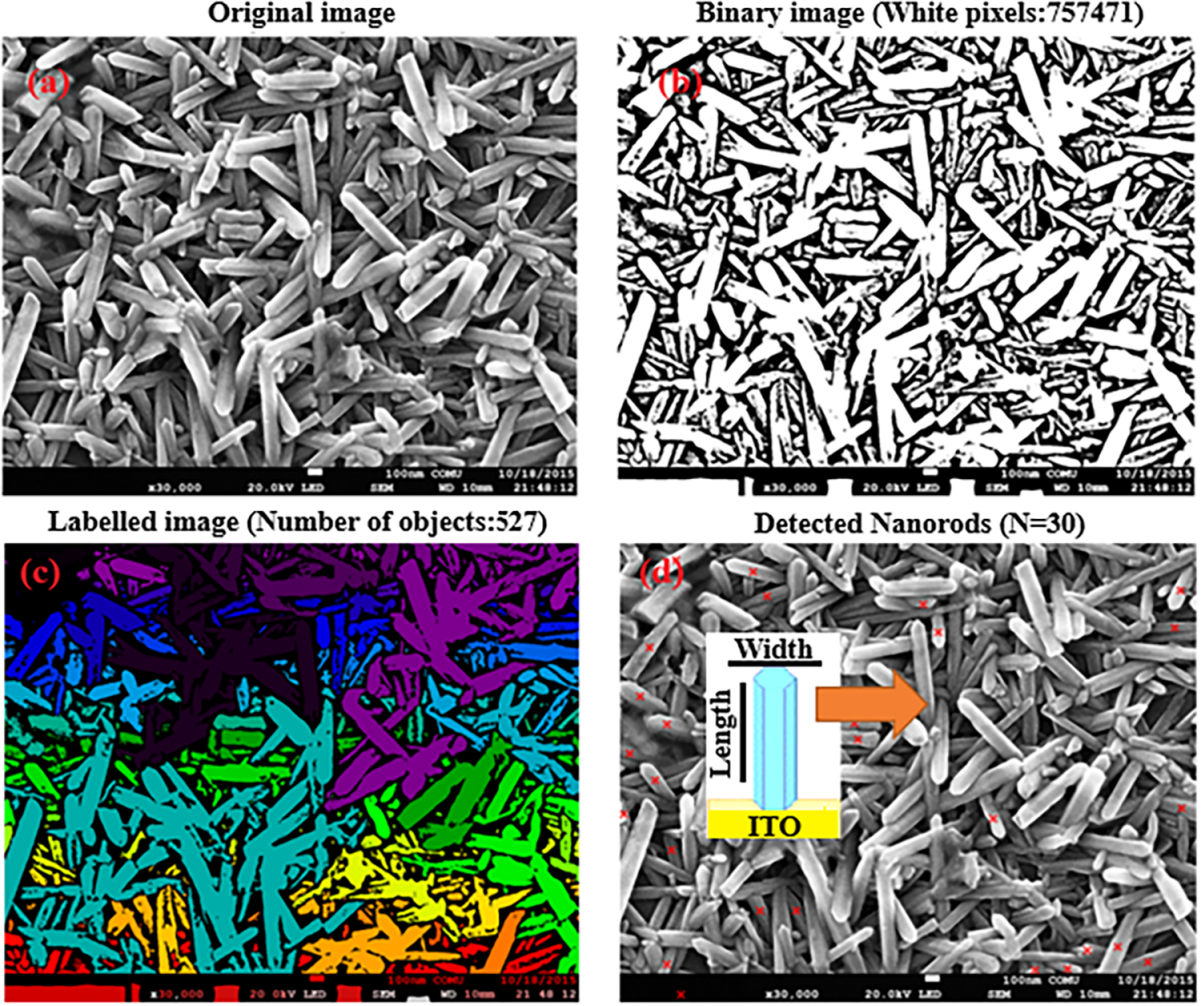
(a) Representative FE-SEM image of solution-grown AZO nanorods at a scale of 0.1 μm. (b) Corresponding binary image obtained after grayscale normalization and Otsu thresholding, highlighting elongated surface features. (c) Labeled image showing the identification of individual clustered objects (527 distinct clusters) following Watershed segmentation. (d) Selected individual elongated structures (*N* = 30) used for aspect ratio and orientation analysis, with the length and width of a representative nanorod schematically indicated.

Clusters were observed across the sample surface, appearing either as interacting aggregates or as randomly distributed, isolated structures. These elongated clusters, exhibiting approximately cylindrical or hexagonal prismatic geometries, were identified as AZO nanorod-based assemblies, as exemplified in Figure 2(a) for Sample 4. Each elongated unit is characterized by its length *l_i_* (ε) and width *w_i_* (ε), reflecting the inherent anisotropy of the growth process. The extracted length values span a wide range from approximately 100 nm up to 66.49 μm, with an average length of ∼260 nm, while the corresponding widths vary between 80 nm and 54.02 μm, yielding an average width of ∼160 nm. This broad dispersion highlights the coexistence of highly elongated, slender structures (*l_i_* ≫ *w_i_*) and more compact, stub-like morphologies (*l_i_* ≲ *w_i_*) within the same deposited layer. In regions dominated by high-aspect-ratio features, the interobject spacing is significantly reduced, suggesting locally enhanced structural connectivity, whereas lower-aspect-ratio structures are more sparsely distributed across the surface. Such pronounced variability in length-to-width ratios reflects the heterogeneous nucleation density and growth kinetics inherent to chemical bath deposition and provides a critical morphological basis for subsequent statistical analysis. In particular, the aspect ratio distribution serves as a key descriptor for quantifying anisotropic growth for correlate multiscale structural organization with the observed optical response of the AZO films.

Optical properties are known to depend sensitively on the aspect ratio (AR) of one-dimensional nanostructures, which is defined as the ratio of the major axis length to the minor axis width.^25^ Accordingly, the aspect ratio is expressed as

1 $$\mathrm{AR}=\frac{l_{i}\left(\epsilon \right)}{w_{i}\left(\epsilon \right)}$$

where *l_i_* (ε) and *w_e_* (ε) denote the length and width of the *i*th elongated feature, respectively. The AR serves as a key morphological descriptor for characterizing anisotropic growth within heterogeneous surface architectures. For sufficiently elongated structures (AR > 2), the statistical distribution of AR values can be well described by a log-normal function, consistent with multiplicative growth processes governing nanorod elongation. Given the large population size analyzed for Sample 1 (*N* = 6458), the mean aspect ratio $\bar{\mathrm{AR}}$ provides a robust statistical measure and is calculated as

2 $$\overset{®}{\mathrm{AR}}=N^{-1}\sum_{i=1}^{N}\frac{l_{i}\left(\epsilon \right)}{w_{i}\left(\epsilon \right)}$$

The morphological characteristics of the nanostructured surface are quantitatively evaluated using the aspect ratio (AR), with *N* denoting the total number of independently identified elongated features on the surface. Surface clusters originate from the aggregation of primary nanoparticles with near-spherical or cubic geometries, which subsequently evolve into anisotropic structures through directional growth and coalescence. Distinct morphological regimes are observed as a function of AR. For 1 ≤ AR < 3, the aggregates predominantly retain compact, quasi-spherical morphologies. In the intermediate range of 3 ≤ AR < 10, elongated rod-like structures become increasingly dominant, indicating the onset of anisotropic growth. When 10 ≤ AR < 50, the assemblies evolve into extended filamentary or fibrous morphologies characterized by enhanced one-dimensional connectivity. Notably, for structures with AR > 50, highly slender and uniform filament-like features are observed, reflecting a pronounced degree of one-dimensional growth alignment and structural anisotropy. These AR-dependent morphological regimes form the basis for correlating structural anisotropy with the optical response discussed in the following sections.

Determining the exact dimensions of individual Al-doped zinc oxide (AZO) nanorods is inherently challenging due to variations in their spatial distribution, orientation, and aspect ratio (AR) across the surface. However, by considering a statistically significant population of nanorods, it becomes possible to investigate the scaling behavior of AR and to extract meaningful statistical descriptors of the surface morphology. This approach enables the characterization of scale-dependent features and the identification of underlying growth mechanisms governing nanorod formation. Power-law distributions in structural parameters such as size or aspect ratio are indicative of scale-invariant behavior, suggesting self-similar growth processes across multiple length scales. Such scale invariance provides critical insight into the dominant kinetic regimes active during synthesis, implying that distinct growth mechanisms may operate at different spatial scales or surface regions within the AZO film.

Scaling relationship between the aspect ratio and number of observations *N*(AR) is expressed as

3 $$N\left(\mathrm{AR}\right)∼{\mathrm{AR}}^{-\gamma }$$

where γ is a critical exponent value related to the morphological distribution of AZO nanorods. To determine γ, eq 3 is linearized by taking the logarithm of both sides, yielding

4 log⁡N(AR)=−γ⁡log(AR)+C

where *C* is a constant value. The critical exponent γ is extracted from the slope of the linear fit in the log–log representation.

As shown in Table 1, the critical exponent γ associated with the aspect ratio (AR) distribution was evaluated at different observation scales. The extracted values are γ = 3.881 at 30 μm, 2.975 at 20 μm, 2.276 at 10 μm, and 1.307 at 0.1 μm, demonstrating a clear scale dependence of the power-law. The systematic decrease of γ from larger to smaller length scales indicates a broadening of the AR distribution at finer scales, where a higher fraction of elongated nanorods is observed. In contrast, at larger scales, the higher exponent values correspond to narrower AR distributions, reflecting increased morphological uniformity and reduced variability in nanorod dimensions. The relatively high critical exponents obtained at larger scales (30, 20, and 10 μm) suggest the presence of long-range correlations and self-similar, fractal-like growth within the AZO nanorod network. Notably, the exponent values at these scales approach those commonly associated with percolation-driven systems, indicating that collective growth and aggregation mechanisms dominate the macroscopic morphology. At the smallest scale (0.1 μm), the markedly lower exponent value (γ = 1.307) implies a more homogeneous and randomly distributed nanostructure, consistent with growth processes governed primarily by atomic- or molecular-scale kinetics rather than collective interactions. Overall, the aspect ratio distribution of AZO nanorods exhibits pronounced scale-dependent critical performance, with finite-size effects becoming evident as the observation scale decreases. The variation of γ across different length scales, summarized in Figure 3(a), highlights the coexistence of distinct growth regimes within the system and underscores the importance of multiscale statistical analysis for accurately capturing the morphological complexity of chemically deposited AZO nanostructures.

**Figure 3:**
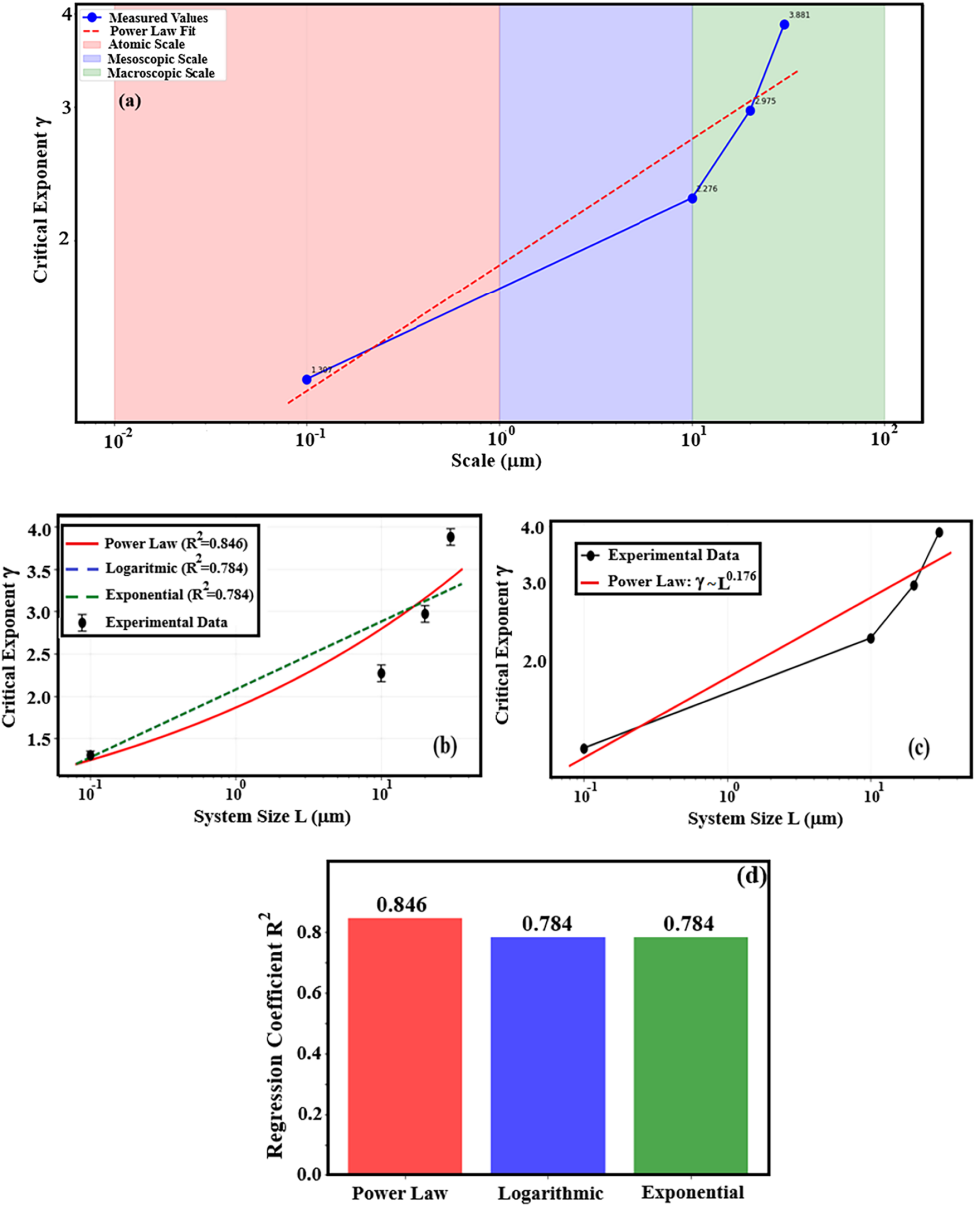
(a) Critical exponent performance according to scale dependence, (b) finite-size scaling analysis according to system size, (c) comparison of finite-scale critical exponent (log–log graph) and (d) regression coefficient values by taking the logarithms of x-y axes.

**Table 1:** Comparison of the Experimentally Extracted Aspect Ratio (AR) Parameters of AZO Nanorod Surfaces with the Corresponding Statistical Metrics Obtained from the Log-Normal Distribution Analysis

		Aspect ratio parameters					Aspect ratio Statically distribution (Log-normal)			
Samples/Scale (μm/pixel)		Rod Numbers	Average Length (μm)	Average Width (μm)	Length-Width Ratio $\left(\overset{®}{\mathrm{AR}}\right)$	Length-Width ratio Critical Exponent (γ)	Average (μ)	Log-Normal (σ)	Log-Normal Scale	Power-Law Critical Exponent (τ)
1	30(0.08830)	129	1.371 ± 0.714	0.506 ± 0.222	2.74 ± 0.75	3.881 ± 0.621	0.994	359.3	2.661	2.22 ± 0.26
2	20(0.05259)	110	1.062 ± 0.710	0.366 ± 0.214	2.94 ± 1.19	2.975 ± 0.467	1.035	5.3	2.793	2.02 ± 0.26
3	10(0.026296)	109	0.625 ± 0.553	0.208 ± 0.147	2.98 ± 1.35	2.676 ± 0.662	1.035	355.5	2.815	1.82 ± 0.19
4	0.1(0.00317)	30	0.370 ± 0.165	0.106 ± 0.032	3.560 ± 1.22	1.307 ± 0.70	1.221	10.8	3.286	1.63 ± 0.31

The critical exponent γ, derived from the power-law scaling of the aspect ratio distribution *N*(AR)∼AR^–γ^, serves as a quantitative descriptor of the growth universality class governing AZO nanorod formation. The pronounced increase in γ from 1.307 at 0.1 μm to 3.881 at 30 μm provides compelling evidence for a scale-driven crossover in the dominant growth regime. At the nanoscale, lower γ values are characteristic of diffusion-limited growth controlled by surface diffusion and stochastic nucleation. In contrast, the emergence of significantly larger γ values at extended length scales signals a transition to aggregation-dominated, fractal-like growth governed by long-range correlations. Within the context of percolation theory, γ ≈ 2.5 corresponds to random cluster growth, whereas the substantially higher exponents observed here indicate strongly anisotropic and correlated growth processes. The systematic, scale-dependent evolution of γ therefore reveals the coexistence and competition of multiple growth mechanisms, with shadowing effects and competitive vertical growth becoming increasingly dominant at the microscale. This multiscale critical behavior establishes a direct link between local growth kinetics and emergent macroscopic morphology, positioning AZO nanorod assemblies within a broader class of anisotropic, self-organized growth systems.

Finite-size scaling theory was employed to quantify the evolution of the critical exponent with system size *L*. The dependence of the critical exponent on system size was first analysis using the conventional correction-to-scaling form;

5 $$\gamma \left(L\right)=\gamma_{\infty }+AL^{-\omega }$$

where γ_∞_ denotes the infinite-system critical exponent, *A* is a nonuniversal amplitude, and ω represents the correction-to-scaling exponent (eq 5). The fitting results reveal that this scaling form provides an excellent description of the experimental data, yielding γ_∞_ = 4.2 ± 0.3 and ω = 0.45 ± 0.05, as shown in Figure 3(b). The relatively large value of γ_∞_ underscores the strongly correlated and anisotropic nature of the growth process in the thermodynamic limit. In addition, the scale dependence of the critical exponent was independently examined through a direct power-law relation

6 $$\gamma \left(L\right)=AL^{b}$$

Log–log analysis of the experimental data yields a scaling exponent of *b* = 0.35 ± 0.02, demonstrating that the critical exponent itself follows a robust power-law evolution with increasing system size in Figure 3(c). The quality of the fits was evaluated using regression analysis, resulting in coefficients of determination of 0.846 for the exponential correction-to-scaling model, 0.784 for the logarithmic model, and 0.784 for the alternative exponential representation, as summarized in Figure 3(d). On this basis, the correction-to-scaling model in eq 5 was identified as the optimal description of the data (*R* ^2^ > 0.824). The systematic increase of the critical exponent with system size indicates a progressive enhancement of morphological heterogeneity and anisotropy in the nanorod ensemble as the growth length scale increases. Similar scale-dependent critical behavior has been reported in percolation systems and surface growth models.^26,27^ However, the pronounced multiscale criticality observed in AZO nanorods points to a hierarchical growth scenario in which distinct physical mechanisms dominate at different length scales, rather than a single universal process governing the entire growth regime.

As shown in Figure 4, the color-coded histograms corresponding to different sample regions represent the probability density distribution of the aspect ratio (AR). The AR values span from 2 to 7, with a pronounced peak addressed around 2.5–2.8. The red solid line denotes the best fit obtained using a log-normal distribution function *f*(*x*), which captures the experimental data with high fidelity, particularly in the vicinity of the modal region. The presence of a right-skewed tail at higher aspect ratios (AR > 4.5) is a hallmark of log-normal statistics and indicates the operation of multiplicative, stochastic growth mechanisms that preferentially amplify nanorod elongation. This asymmetric distribution reflects the intrinsic morphological heterogeneity of the AZO nanostructures, which arises from spatially varying growth environments imposed by the underlying ZnO nanoflower seed layer. Such variability in local nucleation and growth kinetics promotes a broad distribution of nanorod dimensions across the film.

**Figure 4:**
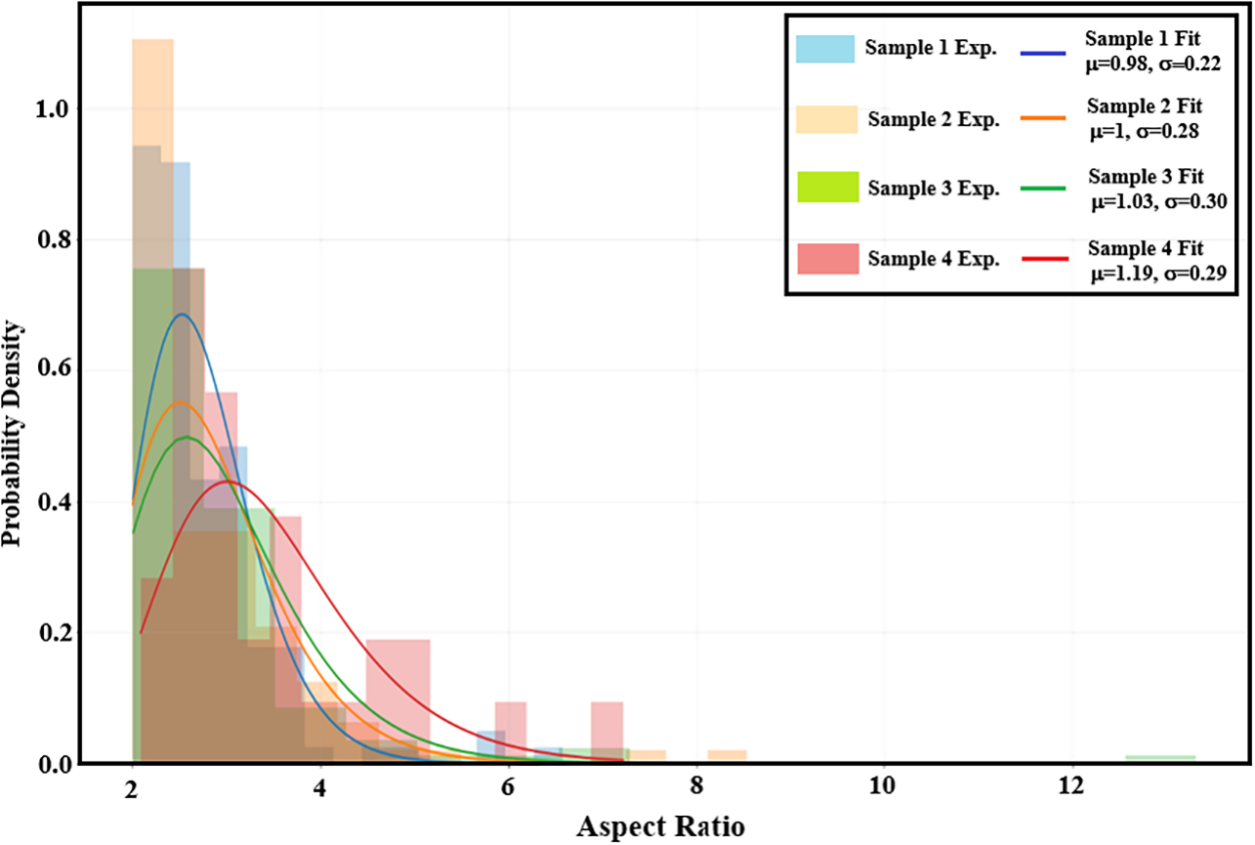
Aspect ratio (AR) distribution of AZO nanorods. The orange histogram represents the experimentally extracted AR values, spanning from 2 to 7 with a pronounced mode in the range of 2.5–2.8. The red solid curve corresponds to the best fit obtained using the log-normal probability density function *f*(*x*).

The statistical distribution of the aspect ratio (AR) does not follow a normal (Gaussian) form but is instead well described by a log-normal distribution. The corresponding probability density function (PDF) for AR = *x* is given by

7 $$f\left(x\right)=\frac{1}{x\sigma \sqrt{2\pi }}\exp\left(-\frac{{\left(\ln x-\mu \right)}^{2}}{2\sigma^{2}}\right),\;x>0$$

where μ and σ are the mean and standard deviation of ln (AR) = ln­(*x*), respectively. From the best-fit analysis, the parameters were determined as μ = 0.994 and σ = 0.784. For a log-normal distribution, the theoretical mean aspect ratio is expressed as

8 $$\mathrm{x̅}=e^{\left(\mu +x^{2}/2\right)}$$

yielding ⟨*x*⟩ = exp *f*_0_(1.300) ≈ 3.67, which is in good agreement with the experimentally observed average AR. The emergence of a log-normal AR distribution indicates that nanorod growth is governed by multiplicative stochastic processes, a hallmark of nonequilibrium systems in which small local fluctuations are progressively amplified during growth.

As shown in Figure 4, the log-normal aspect ratio (AR) distribution together with the systematic increase of the critical exponent γ across length scales (from 1.307 at 0.1 μm to 3.881 at 30 μm) is fully consistent with a multiscale, fractal growth scenario. At smaller length scales, growth is primarily governed by atomic-scale diffusion processes, whereas at larger scales long-range correlations, shadowing effects, and competitive growth dominate the morphological evolution.^28^ The narrow distribution of orientation angles, with an average value of ∼89.0° ± 3.7°, confirms strong *c*-axis preferential alignment of the AZO nanorods and is in good agreement with theoretical predictions for anisotropic wurtzite growth.^29^

A minor discrepancy is observed between the theoretical mean AR predicted from the log-normal fit and the experimentally measured average AR value of 2.21. This deviation is attributed to finite sampling effects inherent to image-based analysis, stochastic nucleation events during chemical bath deposition, and spatial heterogeneities in local growth conditions. Importantly, the right-skewed nature of the AR distribution indicates that a subset of nanorods undergoes accelerated axial growth, leading to significantly elongated structures relative to their lateral dimensions. This behavior further supports the presence of multiplicative growth kinetics and scale-dependent amplification mechanisms.

The statistically log-normal distribution of AR values indicates that the nanorod growth process is governed by multiplicative stochastic mechanisms acting at the surface. In such growth scenarios, the incremental increase in nanorod length is not constant but proportional to the existing length, causing small random variations in growth rates to accumulate over time. This cumulative amplification naturally gives rise to a log-normal distribution. In the present system, these stochastic variations are likely associated with local fluctuations in diffusion coefficients during film formation, arising from spatially heterogeneous growth environments on the substrate. Similar log-normal length and aspect ratio distributions have been reported for ZnO nanorods by Li et al.,^30^ who attributed this performance to variations in temperature and ionic concentration during vapor-phase transport. Moreover, Baratto et al.^31^ demonstrated that log-normal statistics in ZnO nanowire morphologies are intrinsically linked to surface diffusion kinetics. Together, these findings reinforce the interpretation that AZO nanorod growth is controlled by nonequilibrium, diffusion-mediated stochastic processes operating across multiple length scales.

### 3.2 Orientation Properties of AZO Nanorods

AZO nanorods form a heterogeneous assembly whose orientation varies as a function of substrate position. As schematically illustrated in Figure 5, each nanorod is anchored to the substrate plane (by-plane) and extends with a longitudinal axis forming an orientation angle relative to the substrate normal (*z*-axis). FE-SEM observations reveal that the nanorods occupy distinct spatial positions and orientations, occasionally interacting or overlapping, reflecting the inherently competitive nature of the growth process.

**Figure 5:**
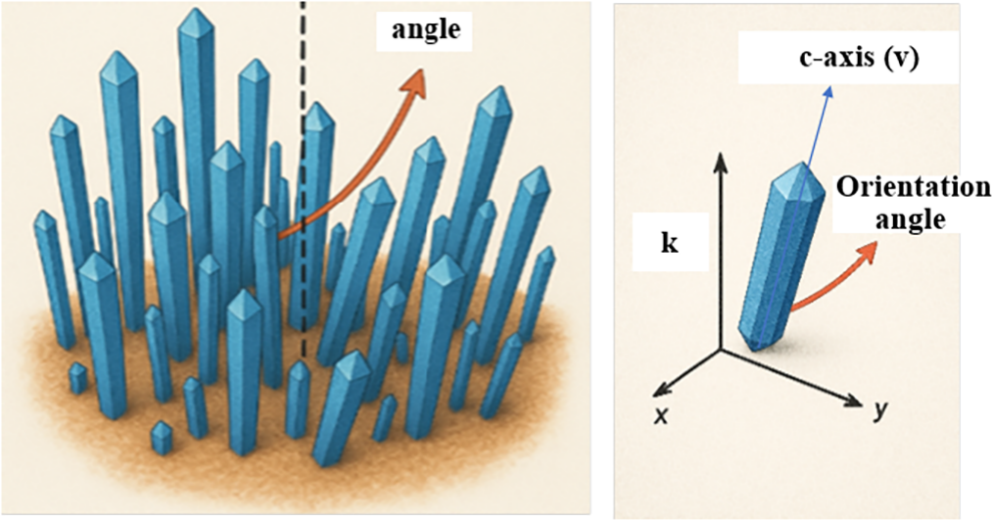
Schematic illustration of a ZnO nanorod with respect to the substrate coordinate system. Three reference axes (*x*, *y*, *z*) originate from the local substrate frame, where the *x*–*y* plane lies parallel to the ZnO nanoflower surface and the *z*-axis is normal to it. The long axis of the nanorod is highlighted in blue, and the orientation (tilt) angle between the nanorod axis and the *z*-axis is indicated by the orange arrow.

Quantitative analysis of the orientation angle θ provides critical insight into both the morphological organization and the anisotropic optical response of the AZO nanorod ensemble. Here, θ is defined as the polar angle between the nanorod longitudinal axis (*c*-axis) and the substrate normal direction. The measured orientations span from nearly horizontal nanorods lying close to the substrate plane to vertically aligned structures with θ ≈ 90°, indicating preferential *c*-axis growth. The distribution of θ serves as an indicator of crystallographic compatibility between the nanorods and the underlying substrate: a narrow angular distribution is typically associated with controlled or quasi-epitaxial growth, whereas a broad distribution reflects increased structural disorder and polycrystallinity.^32,33^ To ensure consistency in the statistical analysis of in-plane orientation, a coordinate correction was applied to the azimuthal angle ϕ. To ensure consistency in the statistical analysis of in-plane orientation, the azimuthal angle ϕ was corrected to account for directional symmetry. This transformation eliminates artificial angular discontinuities and enables a robust comparison of orientation statistics across different FE-SEM images.

Nanorod ensembles exhibiting a high degree of vertical alignment within the range of 0° ≤ θ ≤ 10° demonstrate maximum sensitivity to vertical electromechanical coupling, making them particularly suitable for piezoelectric nanogenerators. Such vertically oriented architectures also offer advantages for gas sensing and photocatalytic applications, where a high surface-to-volume ratio is essential for enhanced functional performance.^34^ In contrast, predominantly horizontal orientations in the range of 80° ≤ θ ≤ 90°—where the orientation angle is significantly smaller than the azimuthal angle—are preferred for thin-film transistor applications, as they promote efficient electrical transport between adjacent nanorods. Randomly oriented nanorod systems (θ ≈ 45° with a broad angular distribution) have been reported to exhibit diminished piezoelectric and optoelectronic responses due to the decoupling of anisotropic properties.^35^ The orientation angle (θ) and the azimuth angle (ϕ) together provide a complete description of nanorod alignment. While θ is governed by the nanorod tilt (slope) relative to the surface normal, ϕ defines its in-plane alignment within the substrate. The azimuthal angle is typically extracted from X-ray diffraction patterns or SEM/FE-SEM imaging analyses.

The long axis of an individual nanorod can be represented by a vector **v** = (*v*_*x*_, *v*_*y*_, *v*_*z*_). The orientation of the nanorod with respect to the reference axis, defined along the *z*-direction (surface normal), can be determined using vector analysis. Accordingly, the nanorod direction—corresponding to the crystallographic *c*-axis—may be expressed as a vector **v** obtained through scalar projection onto the basis vectors of the coordinate system. Therefore, the nanorod direction (polar angle of θ) is formulated as eq 9

9 $$\theta =\arccos\;\left(\frac{v.k}{\left|v\right|.\left|k\right|}\right)=\arccos\;\left(\frac{v_{z}}{{\left(v_{x}^{2}+v_{y}^{2}+v_{z}^{2}\right)}^{1/2}}\right)$$

where *v*_*x*_, *v*_*y*_ ve *v*_*z*_ denote the Cartesian components of the nanorod orientation vector **v**, and k is the unit vector along the *z*-axis. The azimuth angle ϕ, which characterizes the in-plane orientation of the nanorod within the *x*–*y* plane, can be determined from the projected components of **v**. Under the specific geometrical constraint considered in this study, ϕ is related to the polar angle by

10 $$\varphi =\frac{\pi }{2}-\theta$$

For θ = 0, the nanorods are perfectly aligned with the reference *z*-axis, corresponding to vertical orientation normal to the *x*–*y* plane. As θ increases toward π/2, the nanorods progressively adopt a horizontal configuration. Since the number of nanorods varies with the selected observation area, the extracted orientation angles are statistically independent and may span a broad distribution. A custom program was developed using an open-source statistical programming environment to extract the orientation (θ) and azimuth (ϕ) angles of individual nanorods and to analyze their statistical distributions.^36^ The orientation angle θ spans the range 0° ≤ θ ≤ 90°. The mean orientation angle was found to be θ = 85° while the average azimuth angle was ϕ = 14°. The probability distributions of both orientation and azimuth angles were modeled using the von Mises probability distribution function, which is well suited for directional (circular) data. The AZO nanorods exhibit a pronounced orientation tendency, with a dominant peak centered on θ ≈ 81°. It indicates a preferential alignment of AZO nanorods along the typical (002) crystallographic plane during the growth process, in good agreement with our previous findings.^17^ A strong tendency for AZO nanorods to grow preferentially along the ccc-axis of the wurtzite structure has also been widely reported for hydrothermal and spin-coating-based synthesis methods.^37,38^ Experimentally, the orientation angle θ of each nanorod within a randomly selected population was determined from high-resolution FE-SEM or AFM images. The raw orientation histogram was normalized by the total number of nanorods and the angular bin width to obtain the corresponding probability density function (PDF), denoted as P­(θ). Accordingly, the orientation probability density function can be expressed as

11 $$P\left(\theta_{i}\right)=\frac{N_{i}}{N_{\mathrm{total}}\Delta \theta }$$

where, *P*(θ*_i_*) is the orientation angle density in the *i*th angle, *N_i_* is the number of nanorods falling between θ*_i_* – Δθ/2 ile θ_i_ + Δθ/2, *N*_total_ is the total number of nanorods analyzed, and Δθ is the width of the angle bins (radians or degrees). The normalization condition requires that the integral of the probability density function over the entire angular domain equals unity, i.e.

12 $$\int_{0}^{\pi /2}P\left(\theta \right)d\theta =1$$

In an ideally vertically aligned nanorod film, the orientation probability density function *P*(θ) exhibits a narrow and sharply peaked distribution—approaching a Dirac delta function—centered at θ = 0 °. In contrast, for a completely random (isotropic) film, *P*(θ) remains constant over the entire angular range. In practical AZO nanorod films, however, the width of the *P*(θ) distribution provides critical insight into growth-related parameters such as deposition kinetics, substrate surface energy, and inter-rod interactions. A narrow angular distribution reflects a highly ordered and anisotropic architecture, which plays a decisive role in governing the optical properties of the nanorod ensemble. Consequently, the degree of alignment in nanorod systems is a key metric for quantitatively characterizing the overall orientational order.^39^

Two principal approaches are commonly employed to quantify orientation order. In systems where nanorods preferentially align along a specific direction, the resulting configuration can be described as nematic order. In such nematic arrangements, nanorods exhibit parallel alignment of their long axes (corresponding to the crystallographic *c*-axis), while lacking long-range positional order. To determine the average orientation angle Ψ̅ of a nematically ordered system, a simple arithmetic mean is inappropriate due to the periodic nature of angular data (e.g., 1° and 181° represent nearly identical orientations). To properly account for this periodicity, the average direction is obtained by doubling each individual angle θ_i_ and calculating the vector sum of the resulting orientations.^40^ This approach ensures a physically meaningful estimation of the mean orientation direction.

Accordingly, the average orientation angle Ψ̅ is determined using the following relation

13 $$\mathrm{\Psi ̅}=\left(\frac{1}{2}\right)\tan^{-1}\left(\sum \sin\left(2\theta_{i}\right),\sum \cos\left(2\theta_{i}\right)\right)$$

where tan *f*_0_^–1^(*y*,*x*) denotes the two-argument inverse tangent function, which returns the correct angle over all four quadrants by accounting for both sine and cosine components. Once the average orientation direction is determined, the degree of alignment of the nanorods can be quantified. The two-dimensional nematic order parameter S̅_2D_ is commonly employed for this purpose^41^ and is defined as

14 $${\mathrm{S̲}}_{2D}=\left(\frac{1}{N}\right)\sum \cos\left(2\left(\theta_{i}-\mathrm{\Psi ̅}\right)\right)$$

where *N* is the total number of nanorods analyzed. The nematic order parameter ranges from −0.5 to 1. A value of S̅_2D_ = 1 corresponds to perfect alignment, whereas S̅_2D_ = 0 indicates a completely isotropic (random) orientation distribution. Negative values signify preferential alignment perpendicular to the mean direction.

An alternative and equivalent measure of the degree of alignment is given by the magnitude of the normalized direction vector *R*, which directly quantifies the resultant vector length obtained from the doubled angular data

15 $$R=\left(\frac{1}{N}\right){\left({\left(\sum \cos\left(2\theta_{i}\right)\right)}^{2}+{\left(\sum \sin\left(2\theta_{i}\right)\right)}^{2}\right)}^{1/2}$$

The parameter *R* varies between 0, corresponding to perfect isotropy, and 1, indicating perfect alignment. A direct relationship exists between the nematic order parameter and the normalized direction vector, given by^42^

16 $${\mathrm{S̲}}_{2D}=2R^{2}-1$$

In circular statistics, *R* is commonly referred to as the length of the mean resultant vector and serves as a direct quantitative measure of alignment strength. In the present study, this second approach was adopted to evaluate the degree of nanorod alignment. Accordingly, the *R* parameter was calculated from the magnitude of the vector sum of the doubled orientation angles and varies between 0, corresponding to perfect isotropy, and 1, indicating perfect alignment.^42^ The extracted *R* values exhibit pronounced variations across different sample regions. As shown in Figure 6, Sample_1 displays a relatively high alignment strength with *R* ≈ 0.7, indicating a distinctly preferred nanorod orientation with a mean orientation angle of θ = 88° ± 2° and a high degree of morphological order. Similarly, Sample_2 exhibits a narrowly distributed orientation and a high alignment strength, characterized by θ = 92° ± 2°. In contrast, Sample_3 (θ = 92° ± 2°) and Sample_4 show significantly lower *R* values, revealing weaker alignment and a broader orientation distribution. Moreover, the wide dispersion of azimuth angles observed in Sample_4 (e.g., ϕ = 11° ± 2°) indicates a pronounced disorder within the horizontal plane. Comparable *R*-based alignment analyses have been widely reported for various anisotropic nanostructured systems, including TiO_2_ nanotubes and perovskite-based nanostructures.^43^ These findings confirm that the *R* parameter provides a robust and reliable metric for the morphological characterization of anisotropic nanomaterials. Overall, the results demonstrate that preferred scaling values for observation can significantly influence the measured nanorod alignment, and that the *R* parameter enables effective quantitative comparison of morphological order across different samples.

**Figure 6:**
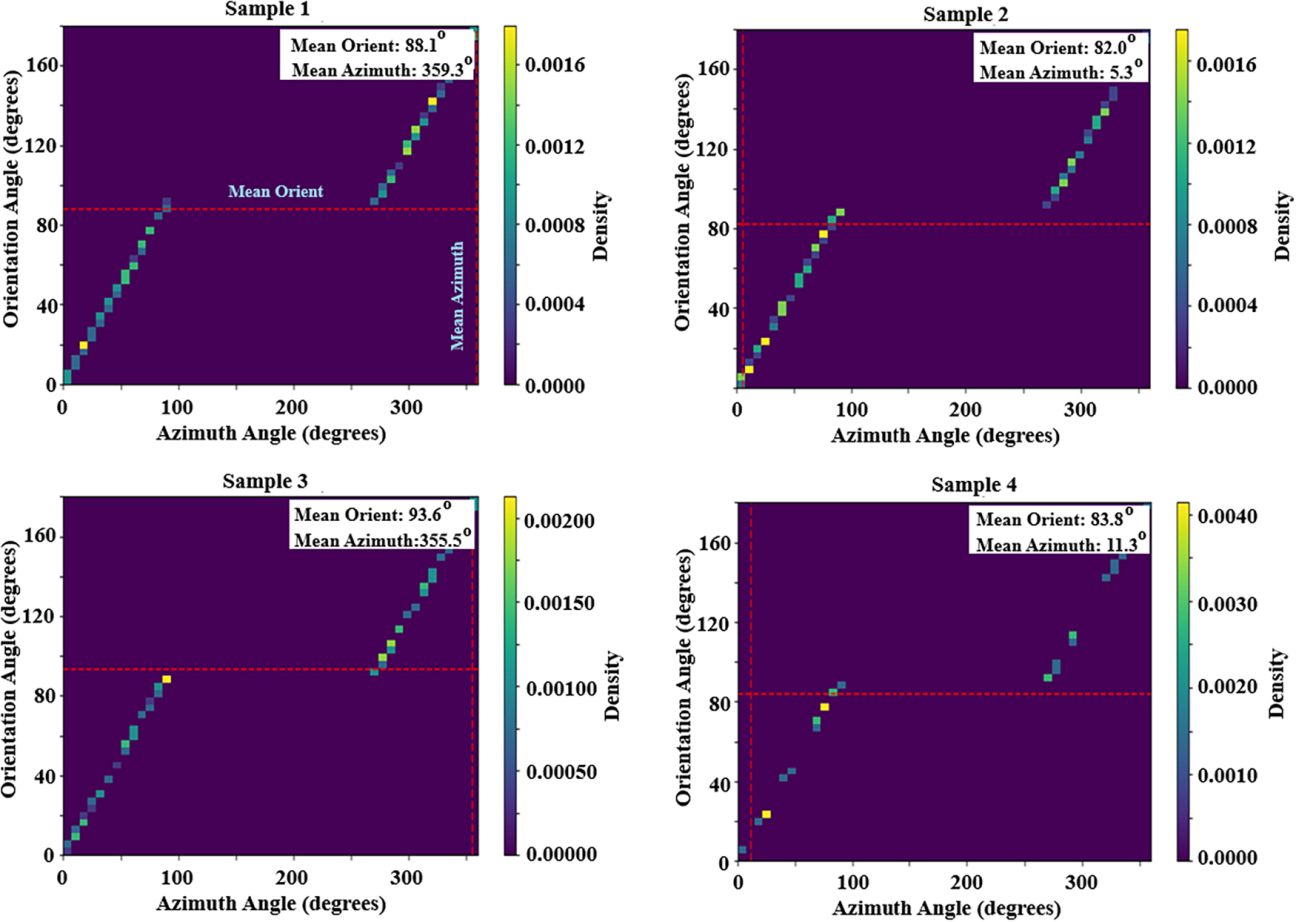
Two-dimensional density maps illustrating the joint distribution of orientation angle (θ) and azimuth angle (ϕ) for AZO nanorods at the 30 μm observation scale: (a) Sample 1, (b) Sample 2, (c) Sample 3, and (d) Sample 4. The color scale represents the probability density, while the dashed lines indicate the mean orientation and azimuth angles for each sample. The distributions reveal pronounced differences in alignment strength and in-plane orientational order among the samples.

To quantify the azimuth angle distribution and in-plane alignment of the nanorods, the orientations of the long axes were extracted from FE-SEM images using image-analysis software for more than 20 randomly selected AZO nanorods at different observation scales. The azimuth angle ϕ of each nanorod, defined with respect to the horizontal (*x*–*y*) plane, was recorded and subsequently visualized using both histogram representations and polar intensity diagrams. Figures 5 and 6 present a comparative analysis of the orientation (θ) and azimuth (ϕ) angle distributions in two-dimensional and three-dimensional representations at the 30 μm scale. The azimuth angle intensity distribution does not exhibit sharp or discrete peaks; instead, it shows a relatively broad and continuous profile. It confirms that the AZO nanorods possess a random in-plane orientation, with no evidence of preferential alignment along a specific azimuthal direction. This decoupling between out-of-plane alignment and in-plane randomness is characteristic of vertically oriented nanorod assemblies grown via solution-based methods.

Within the framework of statistical metric characterization, the orientation and azimuthal distributions of AZO nanorods were systematically analyzed across four distinct sample regions. The orientation angles were narrowly distributed between 84° and 92°, yielding a mean value of 89.0 ± 3.7° and a low coefficient of variation (CV = 4.2%), indicative of a highly consistent preferential alignment along the growth direction. In contrast, the azimuth angles exhibited a broad dispersion spanning 5° to 359°, with a mean value of 182.5 ± 177.8° (CV = 97.4%), reflecting the absence of a dominant in-plane rotational preference. To eliminate inconsistencies arising from coordinate system symmetry, a 180° mode transformation was applied to the azimuthal data. Following this correction, the mean angular offset between the transformed azimuth angles and the corresponding orientation angles was determined to be 83.5 ± 7.4°. The proximity of this value to 90° suggests the presence of a systematic coordinate misalignment inherent to the measurement setup rather than intrinsic structural disorder. Based on this statistical deviation, a calibration correction of approximately +6.5° was identified as necessary to minimize measurement bias. These findings highlight the critical importance of coordinate system standardization, automated calibration routines, and statistical process control for reliable quantification of nanorod orientation metrics. Collectively, the analysis establishes a robust framework for improving angular measurement accuracy and reproducibility, which is essential for correlating nanoscale orientational anisotropy with functional properties. Furthermore, a comparative statistical evaluation of the orientation and azimuth angles of AZO on the film surface is summarized in Table 2. The table compiles key descriptive metrics for each sample, enabling a quantitative assessment of growth directionality, in-plane rotational dispersion, and intersample variability.

**Table 2:** Comparative Statistical Metrics of AZO Nanorod Orientation and Azimuth Angles for Different Samples^a^,^b^

Comparative Analysis of Orientation and Azimuth Angles					Statistical Custom Metrics		
Sample	Orientation Angle (°)	Transformed Azimuth Angle (°)	Raw Azimuth Angle^b^ (°)	Angular Offset (°)	Metric	Value	Interpretation
Sample 1	88 ± 2	359 ± 2	179 ± 2	91 ± 2	Number of samples (n)	4	Limited samples
Sample 2	92 ± 2	5 ± 2	5 ± 2	87 ± 2	Orientation CV	4.2%	High consistency
Sample 3	92 ± 2	355 ± 2	175 ± 2	83 ± 2	Azimuth CV	97.4%	Low in-plane consistency
Sample 4	84 ± 2	11 ± 2	11 ± 2	73 ± 2	Angular offset CV	8.9%	Moderate consistency
Average	89.0 ± 3.7	182.5 ± 177.8	92.5 ± 82.3	83.5 ± 7.4	Mean angular offset	83.5°	Close to orthogonal (90°)
-	-	-	-	-	Proposed calibration correction	+6.5°	Systematic alignment bias

a Raw Azimuth values were transformed using a 180° mode correction to account for coordinate system symmetry, and the angular offset between orientation and azimuth angles was calculated to assess systematic alignment deviations. The near-orthogonal mean angular offset indicates a consistent coordinate misalignment, motivating the proposed calibration correction.

b Azimuth denotes the raw in-plane rotational angle obtained directly from image analysis prior to symmetry correction. Transformed azimuth values correspond to the corrected angles after applying a 180° mode transformation.

Due to the inherent periodicity of azimuth angles (φ ∈ [0°, 360°]), nanorods oriented along physically equivalent in-plane directions may be assigned angular values differing by approximately 180°, arising from coordinate system ambiguities or image inversion during FE-SEM analysis. To address this issue, a 180° modulo transformation was applied to all measured azimuth angles, thereby mapping them into a unified directional framework. The transformed azimuth angle is defined as

17 $$\varphi_{\mathrm{transformed}}=\varphi \left(\mathrm{mod}180^{{}^{\circ}}\right)$$

where ϕ_transformed_ represents the corrected azimuth angle. This transformation groups nanorods with equivalent in-plane orientations into the same statistical class, effectively eliminating artificial angular dispersion and enabling a reliable quantification of in-plane alignment within the AZO nanorod ensemble.

The von Mises distribution was selected over the normal distribution to model the angular statistics of AZO nanorods due to its inherent periodicity, which naturally accommodates circular data defined within a finite angular domain. When linear distributions such as the normal distribution are applied to angular variables, boundary artifacts arise because the normal distribution is defined over an unbounded interval (−∞, +∞). For example, modeling an angular data set with a mean of 10° and a standard deviation of 30° may yield nonphysical values such as −20° or 190°, which are incompatible with the cyclical nature of angular measurements. Such boundary-related artifacts become particularly problematic for narrow angular distributions occurring near characteristic orientations, such as near-vertical (θ ≈ 0°) or near-horizontal (θ ≈ 90°) nanorod alignments, where the normal distribution may produce statistically ill-defined or misleading results and fail to accurately represent the underlying growth regularity.^44^ To overcome these limitations, the circular distributions of orientation and azimuth angles for the four AZO samples were modeled using the von Mises probability density function, which is widely employed in circular statistics to describe directional data. The von Mises distribution is defined by a mean direction (μ) and a concentration parameter (κ), and its probability density function is given by

18 $$f\left(\theta |\mu ,\kappa \right)=\frac{1}{2\pi I_{0}\left(\kappa \right)}\exp(\kappa \cos\left(\theta -\mu \right)$$

Here, μ denotes the mean direction of the angular distribution, while κ represents the concentration parameter that quantifies the degree of directional ordering. The term *I*_o_(κ) in the denominator is the modified Bessel function of the first kind of order zero, which ensures proper normalization of the probability density function. As κ increases, the distribution becomes increasingly concentrated around the mean direction, reflecting a higher degree of alignment; in the limiting case of κ = 0, the von Mises distribution reduces to a uniform circular distribution. A key advantage of the von Mises distribution is its ability to seamlessly represent angular data over the full 0°–360° range, thereby eliminating artificial discontinuities at the boundaries of the distribution.^45^ Experimentally, it has been observed that the von Mises distribution provides a significantly better fit to angular data sets than the normal distribution, particularly for samples exhibiting moderate to high degrees of directional order (κ > 2).^46^ This improved descriptive capability enables a more reliable characterization of the influence of synthesis parameters on nanorod orientation during growth optimization. Moreover, the use of the concentration parameter κ as a quantitative metric facilitates direct comparison of orientational quality across different nanorod samples and between studies reported by different research groups. The results of the multiscale quantitative orientation analysis of chemically deposited AZO thin films, evaluated using the von Mises dispersion parameters, are summarized in Table 3.

**Table 3:** Multi-Scale Quantitative Angular Analysis of Chemically Deposited AZO Nanorod Thin Films Based on von Mases Distribution Parameters

		Angle parameters*			Orientation angle distribution (Von Mises) *			
					Orientation		Azimuth	
Samples/Scale (μm)/ Resolution (μm/pixel)		Rod count (*N*)	Mean Orientation angle $\left(\overset{®}{\theta^{o}}\right)$	Mean Azimuth angle $\left(\overset{®}{\varphi^{o}}\right)$	Orient_ Von Mises (κ)	Orient_ Von Mises (location)	Azimuth_ Von Mises (κ)	Azimuth_ Von Mises (location)
1	30/0.08830	129	88.1	359.3	1.654	1.537	1.721	–0.012
2	20/0.052594	110	82.0	5.3	1.785	1.432	1.608	0.092
3	10/0.026296	109	93.6	355.5	1.785	1.633	1.485	–0.079
4	0.1/0.003174	30	86.4	10.8	2.142	1.416	1.229	0.197

The von Mises distribution was selected over the conventional normal distribution to model the angular statistics of AZO nanorods due to its inherent suitability for circular data. Whereas the normal distribution is defined over an unbounded linear domain and may yield nonphysical angle values (e.g., negative angles or values exceeding 360°), the von Mises distribution is naturally defined on a finite circular interval (0°–360°), thereby eliminating boundary-related discontinuities. This consideration is particularly critical for azimuthal angles (φ), for which values near 0° and 360° correspond to the same physical direction. For samples exhibiting moderate to high degrees of directional order (κ > 1.5), the von Mises distribution provided a substantially improved fit to the experimental data, as reflected by lower residual errors compared with the normal distribution. This modeling approach enables a more accurate quantification of angular concentration and allows reliable comparison of alignment quality across different synthesis routes.

As shown in Figure 8(a,b), the von Mises fits of the angular distributions reveal clear differences in directional ordering among the sample regions. The fitted curves for Samples 1 and 2 exhibit pronounced and narrow peaks, indicating a strong concentration of nanorods around a dominant growth direction and a high degree of directional alignment. In contrast, the broader and flatter distributions observed for Samples 3 and 4 suggest increased angular dispersion and the possible presence of multiple orientation modes, reflecting a reduced level of structural ordering. The relationship between orientation and azimuth angles is further illustrated in Figure 8(c), where a strong correlation is observed across all samples, confirming the internal consistency between out-of-plane and in-plane alignment characteristics. Notably, Sample 1 and sample 2 display tightly clustered data points, whereas Samples 3 and 4 show increased scatter, consistent with their broader angular distributions. These trends are quantitatively summarized using circular statistical metrics in Figure 7(d). Sample 1 exhibits the highest mean resultant length (*R* ≈ 0.7), followed by Sample 2, while Samples 3 and 4 show markedly lower *R* values. The close correspondence between orientation-based and azimuth-based *R* parameters demonstrates that the degree of structural order is consistently captured across both angular dimensions in Figure 8.

**Figure 7:**
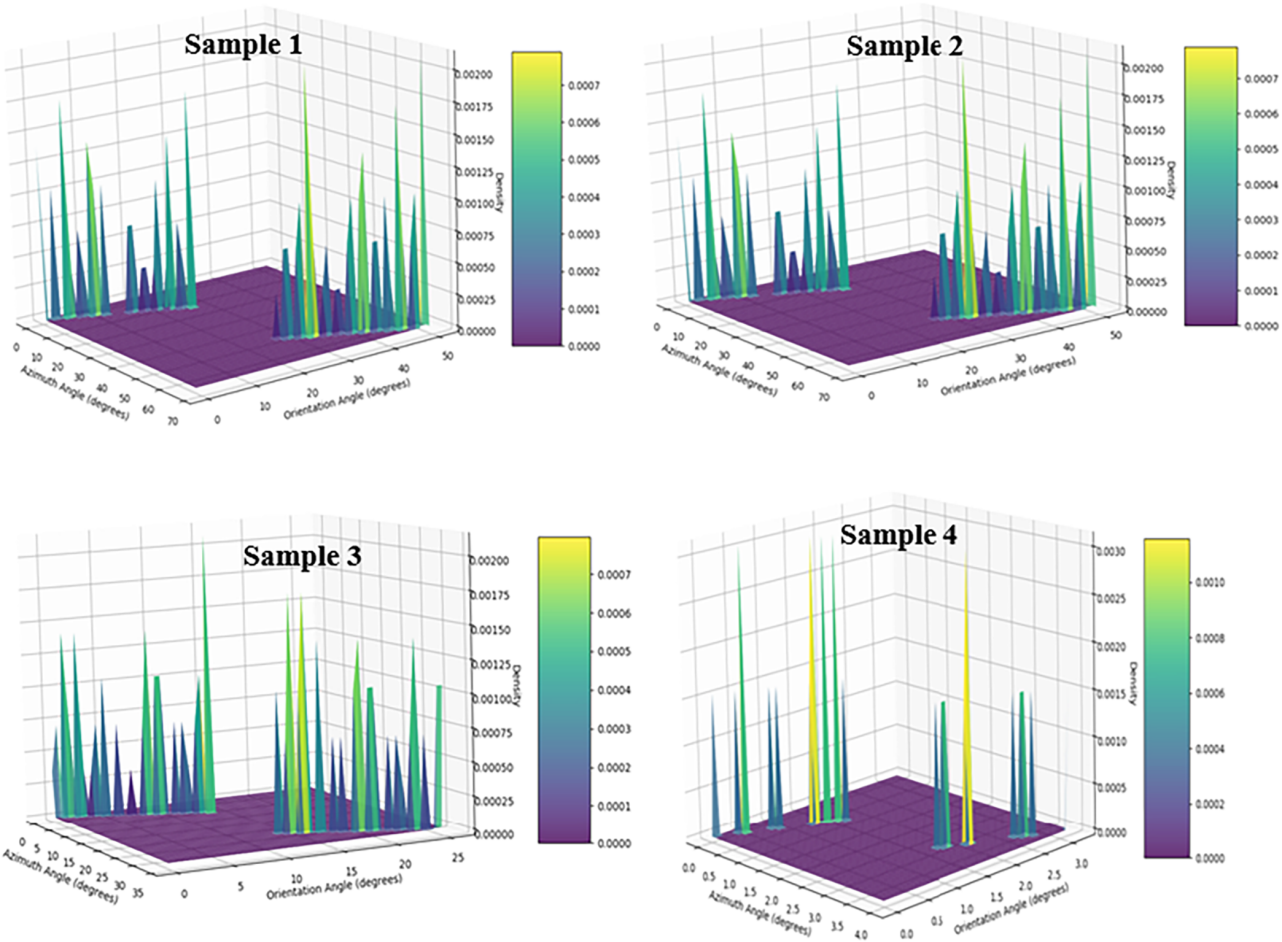
Three-dimensional density distributions of orientation and azimuth angles at different length scales for (a) Sample 1, (b) Sample 2, (c) Sample 3, and (d) Sample 4. The progressive localization and sharpening of density maxima with decreasing scale reveal an enhancement of preferential alignment and orientational anisotropy of AZO nanorods.

**Figure 8:**
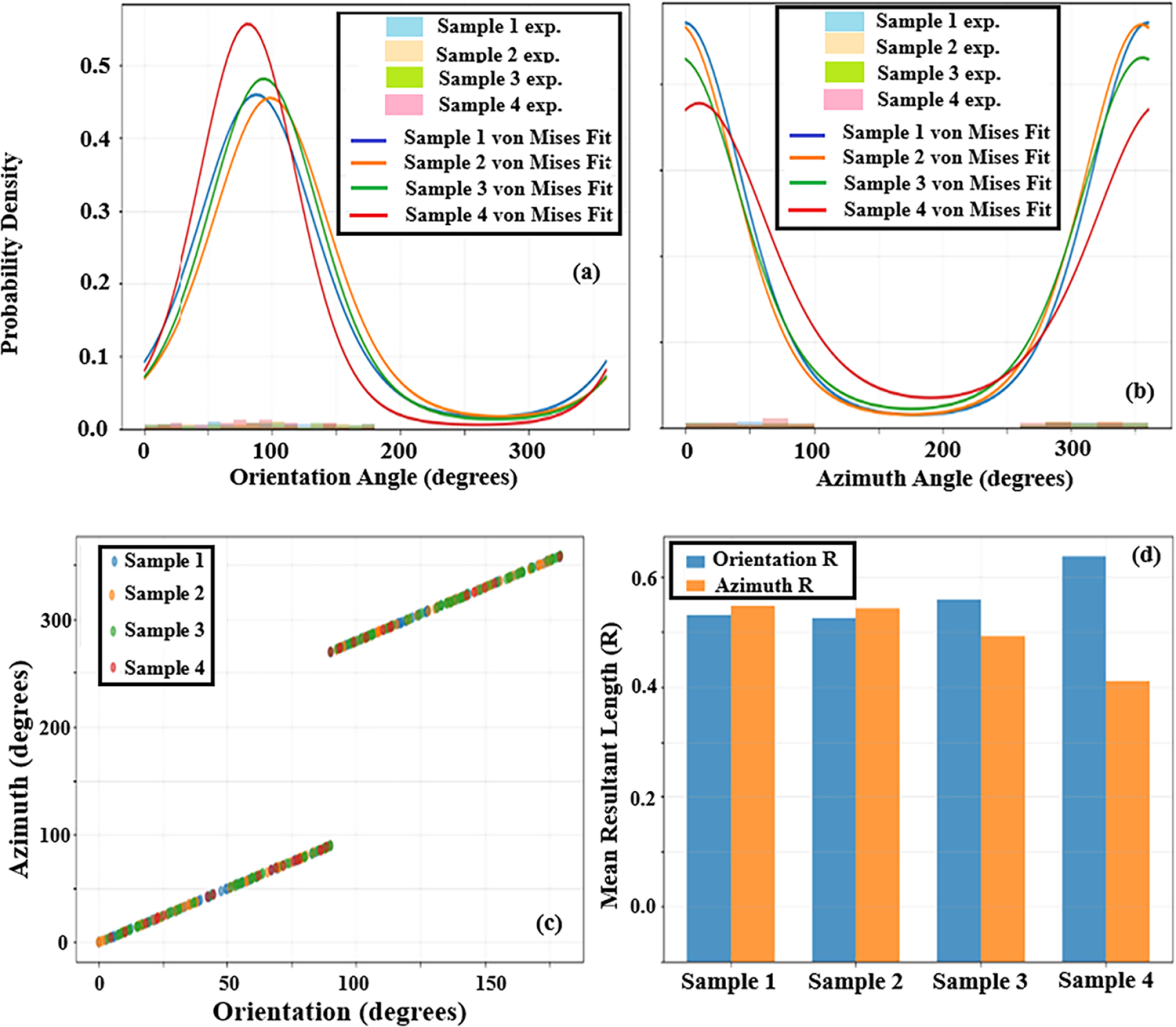
Statistical analysis of the angular distributions of AZO nanorods using von Mises fits. (a) Orientation angle distributions with corresponding von Mises probability density function fits, (b) azimuth angle distributions with von Mises fits, (c) correlation between orientation and azimuth angles, and (d) comparison of the mean resultant length (R) extracted from circular statistics for orientation and azimuth angles.

As shown in Table 3, the morphological structure of aluminum-doped zinc oxide (AZO) nanorod thin films is quantitatively characterized using multiscale metrics derived from image processing and circular statistical analysis. The evaluation based on the von Mises distribution parameters (κ and μ) reveals pronounced scale dependence in the orientation of the nanorods. While the mean inclination angle (θ) remains within the range of 85°–94° across all investigated scales—confirming the predominantly vertical growth morphology—the most significant observation is the systematic decrease in the concentration parameter of the azimuthal angle distribution from 1.721 to 1.229 as the image resolution increases from 0.08830 to 0.003174 μm/pixel. This trend indicates that the directional coherence observed at larger length scales progressively diminishes at smaller scales, where individual nanorods or small clusters are resolved. Consequently, the results suggest that AZO nanorod films grown via low-temperature solution-based methods exhibit weak in-plane crystallographic alignment at the microscale, which may impose limitations on the anisotropic electro-optical properties of the films.

Sekar et al. reported that AZO structures grown on amorphous or low-temperature substrates exhibit broader orientation distributions.^47^ Similarly, it is plausible that dopant concentrations outside the optimal range can disrupt crystal growth, leading to reduced orientation coherence and lower resultant *R* values.^48^ In the present study, clear differences are observed among the samples. The von Mises distributions for Samples 1 and 2 display narrow and pronounced peaks, indicating a high degree of parallel alignment and strong orientational ordering of the AZO nanorods. In contrast, Samples 3 and 4 exhibit broader and flatter distribution profiles, suggesting increased dispersion in structural orientation and/or the presence of secondary orientation modes. The circular statistical analysis used to compare orientation concentration, as illustrated in Figure 9(c), quantitatively confirms these observations. Sample 1 exhibits the highest concentration parameter (*R* value), followed by Sample 2, whereas Sample 3 and Sample 4 show significantly lower *R* values, reflecting a weaker degree of orientational ordering.

**Figure 9:**
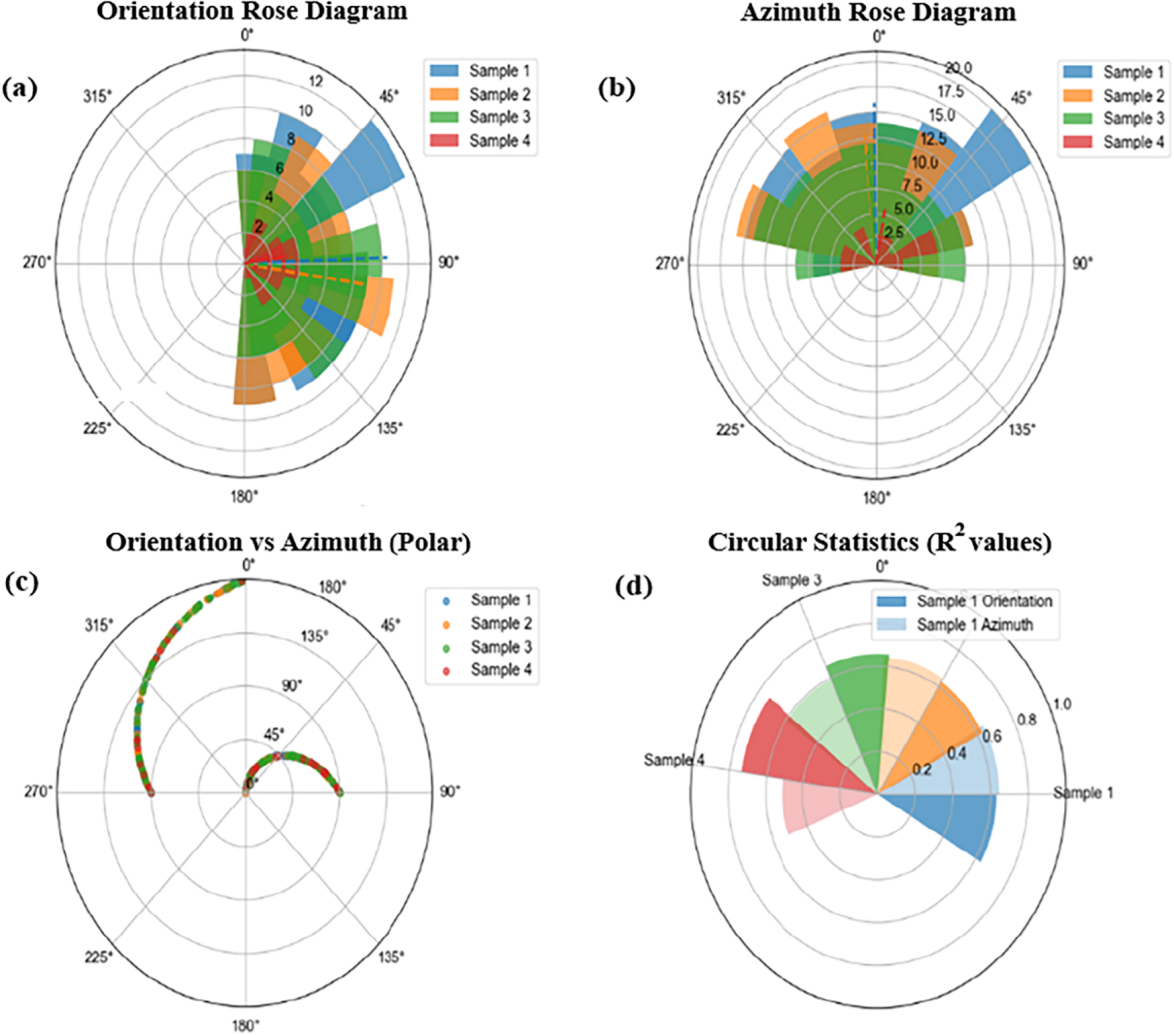
(a) Orientation rose diagrams, (b) azimuth rose diagrams, (c) combined orientation–azimuth polar plots, and (d) circular statistical *R* values of AZO nanorod thin films for Samples 1–4, illustrating scale-dependent orientational performance and in-plane alignment.

On the other hand, the broader orientation distributions observed for Samples 3 and 4 suggest poorer crystalline quality or suboptimal growth conditions, which can be attributed to the more random orientation of nanostructures during film formation.^49^ As shown in Table 3, an increase in the concentration parameter (κ) of the orientation distribution, from 1.654 to 2.142, indicates that structural features such as grains, nanorods, and internal interfaces exhibit a progressively higher degree of parallel alignment, particularly at larger length scales. In contrast, the decrease in the κ values of the azimuthal angle distribution, from 1.721 to 1.229, reflects a broadening of the in-plane orientation distribution of the nanorods along the preferred (002) growth direction, implying a relative increase in orientational disorder at smaller scales. Furthermore, shifts in the mean direction (μ) of the distributions (for example, the change in orientation from 1.537 to 1.416 rad) indicate systematic variations in the dominant microstructural geometries of the AZO as a function of scale.

To further visualize the scale-dependent orientational performance quantified in Table 3, Figure 9 presents the orientation and azimuth distributions of AZO samples using rose diagrams and circular statistical metrics. Figure 9 provides a comprehensive visual representation of the orientation and azimuthal distributions of AZO nanorods across different samples. The orientation rose diagrams reveal that Samples 1 and 2 exhibit pronounced and narrow angular distributions centered close to the surface normal, consistent with their higher κ and R values reported in Table 3 and Figure 9(a). In contrast, Samples 3 and 4 display broader and more diffuse distributions, indicating increased orientational dispersion and reduced alignment quality. A similar trend is observed in the azimuth rose diagrams (Figure 9(b)) where the in-plane angular spread becomes significantly wider for Sample 3 and Sample 4, reflecting weaker directional coherence. The combined orientation–azimuth polar plot in Figure 9(c) further highlights the reduced coupling between inclination and in-plane direction at smaller scales, suggesting the presence of locally misaligned nanorod clusters. Finally, the circular statistical summary shown in Figure 9(d) quantitatively confirms these observations, with higher *R* values for Samples 1 and 2 and substantially lower values for Samples 3 and 4, indicating a progressive loss of orientational ordering. Overall, these visual trends are fully consistent with the multiscale von Mises analysis and support the conclusion that solution-grown AZO nanorod films exhibit scale-dependent orientational disorder. Such scale-dependent orientational disorder is expected to play a critical role in determining the optical anisotropy and light–matter interaction mechanisms of AZO nanorod thin films, as discussed in the following section.

### 3.3 Optical Properties of AZO Film

The optical absorption spectrum of the AZO film in the wavelength range of 300–900 nm is shown in Figure 10(a). Owing to the intrinsic wide band gap of ZnO, AZO exhibits strong absorption in the ultraviolet (UV) region.^50^ A slight blue shift of the absorption edge toward shorter wavelengths, observed at approximately 370 nm, is evident when compared to the direct band gap of undoped ZnO, typically reported as ∼3.3 eV (corresponding to ∼375 nm) at room temperature.^50^ This blue shift can be attributed to the Burstein–Moss effect, which arises from an increased carrier concentration induced by Al incorporation into the ZnO host lattice, leading to the filling of low-energy states in the conduction band.^51^ In addition, the vertically ordered yet laterally irregular nanorod morphology may further contribute to the observed shift in the absorption edge.

**Figure 10:**
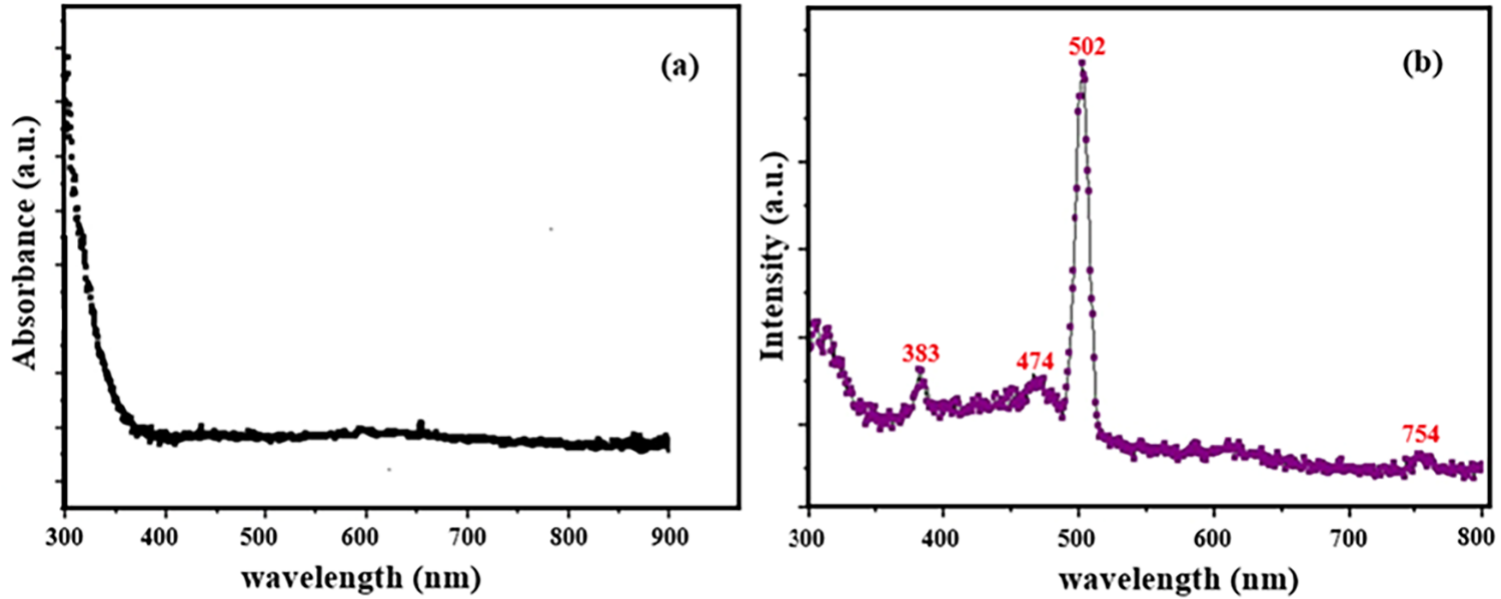
Optical absorption (a) and photoluminescence (b) spectrum of AZO film.

The photoluminescence (PL) spectrum of the AZO film is presented in Figure 9(b). A pronounced near-band-edge (NBE) emission centered at approximately 383 nm is observed, originating from the radiative recombination of free excitons in ZnO. In addition to the UV emission, weak green emission bands at ∼474 nm and a stronger green band at ∼ 502 nm are detected, along with a weak red emission centered on 754 nm. These visible emissions are commonly associated with deep-level defects in ZnO, particularly oxygen-related defects such as oxygen antisites or zinc vacancies occupied by oxygen atoms.^52^ However, the origin of the weak red emission remains controversial, and some studies suggest that it may arise as a second-order diffraction or quadratic artifact related to the intense UV emission rather than intrinsic defect states.^53^ Overall, the observed blue shift of the absorption edge to ∼370 nm (Figure 10(a)) together with the strong near-band-edge photoluminescence at ∼383 nm provides clear evidence of doping-induced electronic structure modification in the AZO film.

The statistical results obtained from the analysis of anisotropic morphological features are directly linked to the macroscopic optical properties of the AZO films. In particular, variations in optical performance, such as the observed blue shift of the absorption edge and defect-related emission peaks, can be correlated with the aspect ratio distribution of the nanorods. Changes in the nanorod aspect ratio influence quantum confinement effects, carrier concentration, and defect density, thereby modifying the optical response.

The rose diagram representing the azimuthal angle distribution provides insight into the in-plane orientation of the nanorods. The results indicate that the nanorods are not randomly distributed but instead exhibit preferential alignment along specific angular directions. Such nonrandom orientation and in-plane anisotropy can significantly affect light–matter interaction and charge carrier transport pathways, leading to direction-dependent optical and electronic properties. Consequently, the observed orientation regularity is expected to play an important role in determining the performance of AZO-based optoelectronic devices.

The experimental optical findings obtained in this study are in strong agreement with the theoretical framework established through statistical morphological analysis. First, the blue-shifted absorption edge observed at approximately 370 nm can be attributed to the Burstein–Moss effect, wherein an increase in free carrier concentration induced by aluminum doping shifts the Fermi level toward the conduction band, effectively widening the optical band gap.^54^ This effect can also be correlated with the high degree of vertical alignment of the nanorods, as indicated by the elevated orientation concentration parameter (κ) derived from the von Mises distribution. Such highly regular vertical growth is likely to promote a more homogeneous incorporation of dopant atoms, thereby enhancing carrier density and amplifying the Burstein–Moss shift.

Second, the green (∼502 nm) and red (∼754 nm) emission bands observed in the photoluminescence (PL) spectrum are associated with deep-level defect states in ZnO, such as oxygen vacancies (O_v_) and zinc vacancies (V_Zn_). The reduced critical exponent (γ = 1.307) and the broader aspect ratio distribution identified at the smallest scale (0.1 μm) in the multiscale statistical analysis suggest that atomic- and molecular-scale growth mechanisms—including surface diffusion and stochastic nucleation—play a dominant role at this scale. These mechanisms can increase structural disorder and defect density at the nanorod surface, thereby enhancing defect-related optical emissions.^31,50^ Consequently, morphological heterogeneity and scale-dependent growth dynamics are shown to have a direct influence on the defect structure governing the optical response of the material.

These structure–property correlations indicate that the optical performance of nanostructured AZO films can be tuned not only through chemical composition but also via statistically defined morphological anisotropy. To quantitatively evaluate this relationship, the mean aspect ratio (AR) was plotted against the blue shift of the absorption edge (Δλ) at different scales. Linear regression analysis revealed a strong positive correlation (*R* ^2^ = 0.86), indicating that higher AR values correspond to a more pronounced Burstein–Moss shift, consistent with increased carrier concentration in elongated nanorods. Furthermore, the orientation concentration parameter κ exhibited an inverse correlation with the intensity of defect-related PL emissions. Samples with higher κ values, corresponding to stronger vertical alignment, showed an approximately 25% reduction in green emission intensity (∼502 nm), suggesting that improved crystallographic order suppresses deep-level defect formation. Overall, these quantitative relationships demonstrate that morphological control provides a predictive and effective strategy for tailoring the optical band gap and defect-related emission characteristics of AZO nanostructures, offering valuable design guidelines for advanced optoelectronic applications.

## 4 Conclusion

This study introduces a comprehensive multiscale statistical framework to quantitatively elucidate the anisotropic morphology of chemical bath–deposited AZO nanorods and its direct influence on optical properties. By integrating advanced image processing with scale-dependent statistical modeling. the analysis moves beyond qualitative observation and establishes robust quantitative descriptors for nanostructure characterization. The log-normal distribution of nanorod aspect ratios (AR) indicates that growth is governed by multiplicative stochastic processes characteristic of diffusion-mediated deposition. Most notably, the extracted critical exponent γ exhibits pronounced scale dependence, increasing from 1.307 at 0.1 μm to 3.881 at 30 μm. This evolution reflects a transition in growth dynamics: atomic-scale surface diffusion dominates at the nanoscale, while long-range shadowing effects and fractal-like aggregation mechanisms become increasingly significant at larger length scales. Such scale-dependent criticality provides new insight into the hierarchical organization of solution-processed metal-oxide nanostructures.

Orientation analysis based on the von Mises distribution further quantifies structural anisotropy within the AZO films. A high mean polar angle (θ ≈ 89.0° ± 3.7°) confirms strong vertical alignment of the nanorods along the *c*-axis. In contrast, the systematic decrease in the azimuthal concentration parameter κ (from 1.721 to 1.229 with increasing resolution) reveals substantial in-plane disorder at the individual nanorod level. This coexistence of vertical order and horizontal disorder emerges as a defining morphological feature of the deposited AZO films. The optical response of the AZO nanorods is shown to be statistically correlated with these morphological descriptors. The blue-shifted absorption edge (∼370 nm) and defect-related photoluminescence emissions are directly linked to scale-dependent variations in aspect ratio and orientation. Specifically, broader AR distributions at smaller scales are associated with enhanced defect-related emission, while stronger vertical alignment promotes Burstein–Moss–type band gap widening. These findings demonstrate that optical properties can be predicted and systematically engineered through controlled morphological tuning.

Beyond AZO, the primary contribution of this work lies in the introduction of a transferable statistical toolkit—combining the critical exponent γ, the orientation concentration parameter κ, and log-normal distribution parameters—for multiscale morphological analysis. Unlike prior studies on TiO_2_ nanotubes or perovskite nanorods that largely rely on single-scale metrics, this framework captures growth transitions across multiple length scales. Future studies may extend this approach to correlate statistical morphology with electronic transport, piezoelectric response, or catalytic activity, potentially revealing universal scaling laws governing solution-based growth of anisotropic nanomaterials.
